# Activity assays of NnlA homologs suggest the natural product *N*-nitroglycine is degraded by diverse bacteria

**DOI:** 10.3762/bjoc.20.75

**Published:** 2024-04-17

**Authors:** Kara A Strickland, Brenda Martinez Rodriguez, Ashley A Holland, Shelby Wagner, Michelle Luna-Alva, David E Graham, Jonathan D Caranto

**Affiliations:** 1 Department of Chemistry, University of Central Florida, Orlando, FL 32816, USA; 2 Biosciences Division, Oak Ridge National Laboratory, Oak Ridge, TN 37831, USA

**Keywords:** enzymology, natural products, nitramine, N–N bond

## Abstract

Linear nitramines (R–N(R′)NO_2_; R′ = H or alkyl) are toxic compounds, some with environmental relevance, while others are rare natural product nitramines. One of these natural product nitramines is *N*-nitroglycine (NNG), which is produced by some *Streptomyces* strains and exhibits antibiotic activity towards Gram-negative bacteria. An NNG degrading heme enzyme, called NnlA, has recently been discovered in the genome of *Variovorax sp.* strain JS1663 (*Vs* NnlA). Evidence is presented that NnlA and therefore, NNG degradation activity is widespread. To achieve this objective, we characterized and tested the NNG degradation activity of five *Vs* NnlA homologs originating from bacteria spanning several classes and isolated from geographically distinct locations. *E. coli* transformants containing all five homologs converted NNG to nitrite. Four of these five homologs were isolated and characterized. Each isolated homolog exhibited similar oligomerization and heme occupancy as *Vs* NnlA. Reduction of this heme was shown to be required for NnlA activity in each homolog, and each homolog degraded NNG to glyoxylate, NO_2_^−^ and NH_4_^+^ in accordance with observations of *Vs* NnlA. It was also shown that NnlA cannot degrade the NNG analog 2-nitroaminoethanol. The combined data strongly suggest that NnlA enzymes specifically degrade NNG and are found in diverse bacteria and environments. These results imply that NNG is also produced in diverse environments and NnlA may act as a detoxification enzyme to protect bacteria from exposure to NNG.

## Introduction

Degradation of nitramines (R–N(R′)NO_2_; R′ = H or alkyl) has been well studied in the context of the environmental degradation of explosive cyclic nitramines [1–2]. The cyclic nitramines hexahydro-1,3,5-trinitro-1,3,5-triazine (commonly called RDX), octogen (HMX), and hexanitrohexaazaisowurtzitane (CL-20) are compounds found in military grade explosives and propellants. Contamination of these cyclic nitramines in soil and groundwater is concerning due to their toxicity and potential carcinogenicity [3–8]. Biotic and abiotic degradation of cyclic nitramines often produce linear nitramine byproducts. For example, degradation of RDX and HMX by microbes or alkaline hydrolysis forms the linear nitramine 4-nitro-2,4-diazabutanal (NDAB) [9–12]. Linear nitramines are also produced during the process of amine-based carbon dioxide capture technologies [13–14]. These linear nitramines from these reactions pose their own health and environmental consequences [13]. Therefore, strategies to remediate linear nitramines are needed.

Compared to cyclic nitramines [1–2,15–16], there is far less known regarding the environmental biodegradation pathways of linear nitramine contaminants. Biodegradations of NDAB by the fungus *Phanerochaete chrysosporium* and the bacterium *Methylobacterium sp.* strain JS178 have been reported [17–18]. Initiation of the *P. chrysosporium* degradation was attributed to a manganese peroxidase, however, the mechanism of degradation is unclear. Linear nitramines, produced by carbon capture, were shown to be biodegraded in soil and water [19]. Nitramines with hydroxy groups were best degraded in this study, including diethylnitramine, 2-methyl-2-(nitroamino)-1-propanol, and 2-nitroaminoethanol (2-NAE). While little is known regarding the degradation of these anthropogenic linear nitramines, we can glean insight into their reactivities from recent studies regarding the enzymatic degradation of *N*-nitroglycine (NNG), a naturally occurring linear nitramine.

**Scheme 1:**
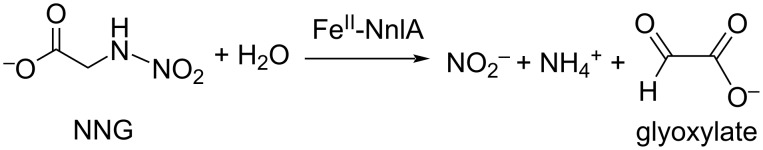
NNG degradation by reduced NnlA.

An enzyme, *N*-nitroglycine lyase A (NnlA), from the bacterium *Variovorax sp.* strain JS1663 (*Vs* NnlA) was recently shown to degrade NNG. This strain was enriched from sludge from the Holston Army Ammunition Plant using selective growth media containing NNG as the only carbon and nitrogen source [20]. The discovery of the *nnlA* gene resulted from screening a JS1663 genomic library by monitoring for *E. coli* transformants that produced nitrite (NO_2_^−^) in the presence of NNG. Analysis of the mass balance via in vitro experiments showed that NnlA degraded NNG into NO_2_^−^, ammonium (NH_4_^+^), and glyoxylate (Scheme 1) [20–21]. *Vs* NnlA contains a Per-Arnt-Sim (PAS) domain – protein domains that often bind heme and function as gas or redox sensors [20]. Indeed, *Vs* NnlA was shown to contain a heme cofactor [21]. Mutagenesis of a predicted histidine ligand to this heme resulted in loss of the heme and the variant could not degrade NNG. Additionally, this heme must be reduced to the ferrous (Fe^II^) state to initiate NNG degradation. Therefore, the heme is critical for NnlA’s NNG degradation activity.

While the activity of NnlA is established less is known about its physiological function and, for that matter, the physiological function of its substrate NNG. This compound is one of the few known nitramine natural products and the only one produced by bacteria instead of fungi [22]. Its only known natural sources are strains of *Streptomyces* bacteria [23–24]. The abundance and distribution of these NNG producers and of NNG is unknown. Additionally, NNG’s physiological function is unknown, but it is toxic to plants, mice, and Gram-negative bacteria [25–26]. While there is no direct evidence of the mechanism of this toxicity, NNG has been shown to competitively inhibit succinate dehydrogenase, a component enzyme of the Krebs cycle [25]. Therefore, NNG may be a toxin released to kill or outcompete nearby bacteria or other organisms for limited resources. In such a context, the physiological function of NnlA could be to protect bacteria from toxic NNG exposure. Alternatively, NnlA could be a promiscuous nitramine degrader that allows bacteria to use alternative nitrogen sources. In fact, the RDX-degrading enzyme, XplA, is remarkably conserved amongst RDX-degrading microbes (>99% identity across several species) [16,27–28]. Based on these observations, it has been proposed that XplA evolved within the past 100 years in response to the rise in RDX contamination. Given that *Variovorax sp.* strain JS1663 was isolated from a nitramine-contaminated sludge, it should be considered if NnlA is promiscuous and can degrade both natural nitramines, such as NNG, and anthropogenic nitramines, such as 2-NAE.

We have previously identified several *Vs* NnlA homologs in sequence databases [20], however, the NNG degradation activities of these homologs have not been tested. Doing so will differentiate between these two hypotheses by testing if NnlA homologs with NNG degradation activity are highly conserved or if they are found in bacteria found in widespread classes and environments. Herein, we report the characterization of five NnlA homologs. It is shown that all five homologs exhibited NNG degradation activity. Isolation and characterization of four of these homologs showed that all contain heme, the reduction of which is required for NNG degradation activity. In addition, we show that NnlA cannot degrade 2-NAE. Combined with previous substrate scope studies, this result strongly suggests that NnlA is specific for NNG. The implications of our results in understanding the environmental abundance and physiological function of NNG are discussed below.

## Results

### Screening of *Vs* NnlA homologs for NNG degradation activity

To select *Vs* NnlA homologs to test for NNG degradation activity, we performed a BLAST search of the *Vs* NnlA amino acid sequence in the NCBI database. This search resulted in retrieval of 99 homologous amino acid sequences with an E-value of less than 1 × 10^−10^. A subset of 55 of these sequences was selected with a query cover of greater than 88%. From these sequences, we selected five homologs of the *nnlA* gene [*Pseudovibrio denitrificans* JCM 1230 (*Pd*), *Pseudovibrio japonicus* strain KCTC 12861 (*Pj*), *Pseudonocardia spinosispora* DSM 44797 (*Ps*), *Mycobacterium sp.* 1465703.*0* (*Ms*)*, Microbispora rosea subsp. nonnitritogenes* strain NRRL B-2631 (*Mr*)], which were synthesized and cloned into *E. coli* recombinant expression vectors. These homologs ranged in amino acid sequence identity from 46 to 76% compared to *Vs* NnlA. Additionally, these homologs along with *Vs* NnlA are found in bacteria that span a wide range of bacterial classes (Alphaproteobacteria, Betaproteobacteria, and Actinomycetia).

A preliminary screen of these homologs for NNG degradation activity was performed to identify homologs for further characterization. The H73A *Vs* NnlA variant, previously shown to lack NNG degradation activity, was used as a negative control [21]. *E. coli* transformants containing the expression vectors were incubated at 37 °C overnight in diluted lysogeny broth (LB) containing NNG and IPTG, the latter component was used to induce NnlA expression.

**Figure 1:**
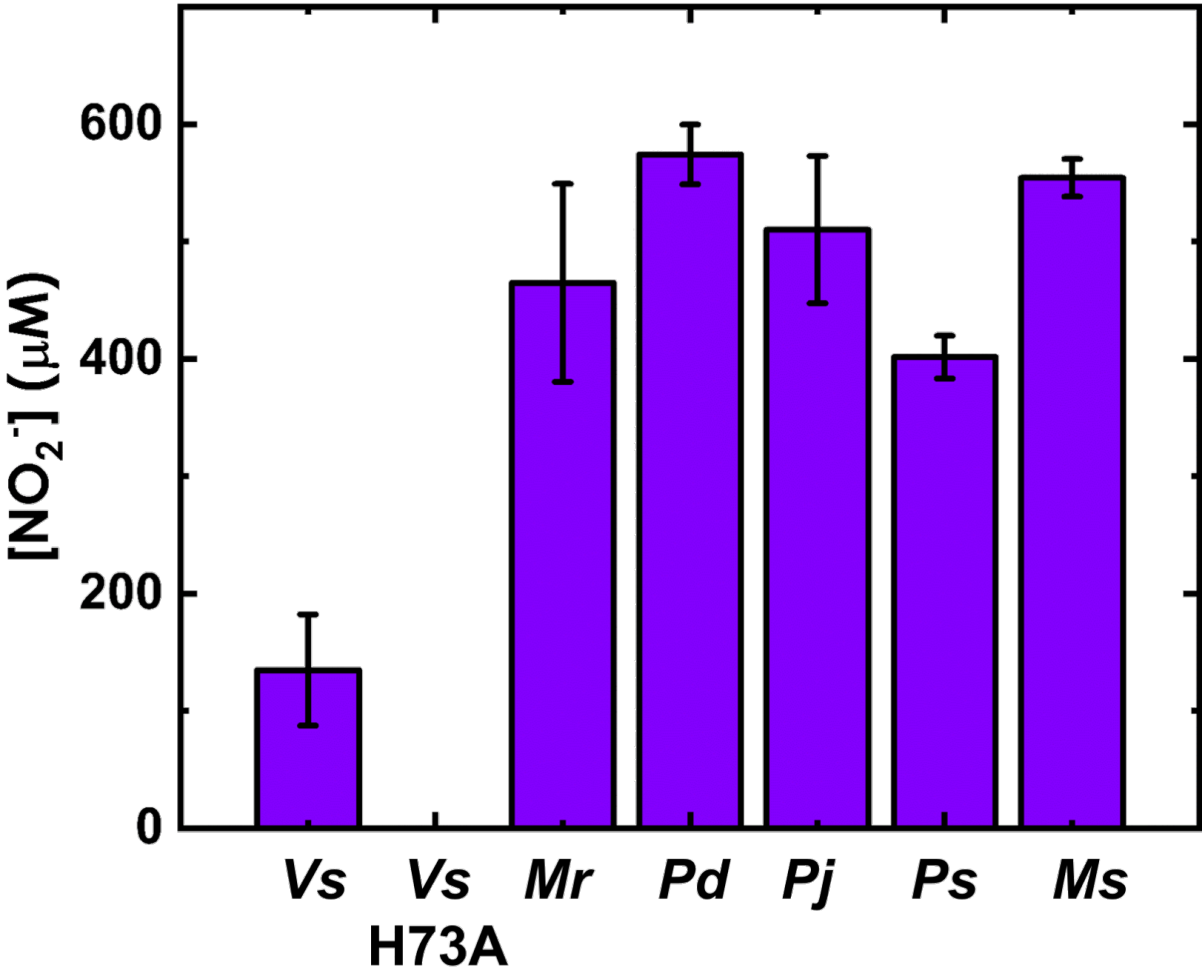
Nitrite concentrations observed in cultures of *E. coli* transformed with NnlA homologs or variants grown in the presence of NNG. Cells were incubated overnight in 1/5 LB, 20 µM IPTG, 3 mM NNG with appropriate antibiotics, incubated overnight at 37 °C.

Overnight cultures of *Vs* NnlA and all five of the selected homologs exhibited nitrite formation as measured by the Griess assay (Figure 1). By contrast, cultures expressing H73A *Vs* NnlA lacked NO_2_^−^. This result strongly suggests that the NO_2_^−^ observed in the experimental samples resulted from NNG degradation activity by the recombinantly expressed NnlA homologs. We conclude from these results that all five of the selected NnlA homologs exhibit NNG degradation activity.

### NnlA homologs exhibit similar heme and iron occupancy as *Vs* NnlA

**Figure 2:**
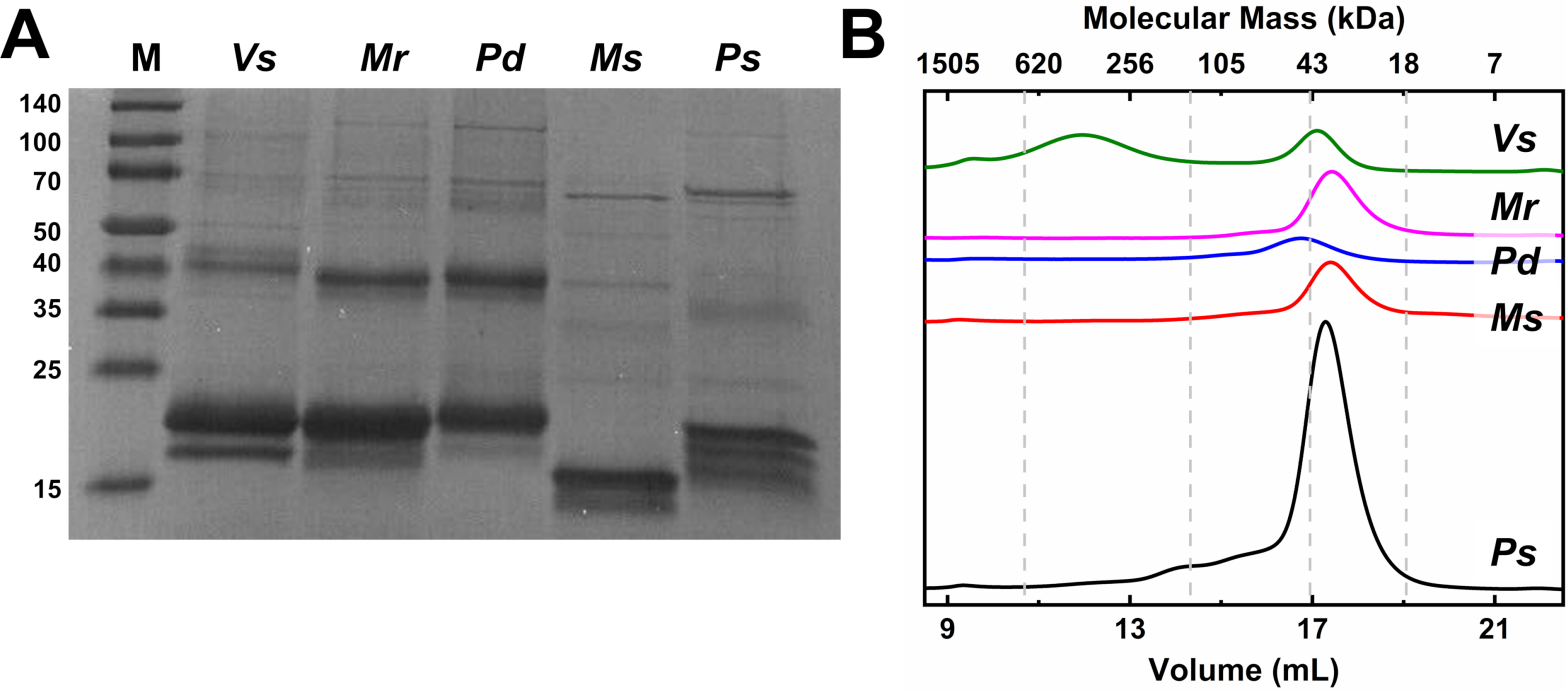
Molecular mass determination of purified NnlA homologs by A) SDS-PAGE or B) analytical size exclusion chromatography. Homolog labeled in figure. Mobile phase and sample buffer is 100 mM tricine 100 mM NaCl buffer at pH 7.5. Dashed grey lines represent elution volumes of molecular mass standards. Theoretical molecular weights are as follows *Vs* NnlA: 21,869 Da; *Mr* NnlA: 20638 Da; *Pd* NnlA: 18,473 Da; *Ms* NnlA : 18,239 Da; and *Ps* NnlA: 19,042 Da.

To better compare the NnlA homologs to *Vs* NnlA, each homolog was recombinantly expressed in *E. coli* and purified. One of the five homologs, *Pj* NnlA, was found to be substantially insoluble and thus, could not be isolated. The remaining four homologs were isolated by immobilized metal affinity chromatography. While the most prominent band observed in the SDS-PAGE gel of each homolog is consistent with the expected monomer molecular masses of approximately 21 kDa, several other bands appear (Figure 2A). The banding patterns of each of the homologs are similar to those of *Vs* NnlA. These higher molecular weight bands are likely not contaminants but are either undissociated higher oligomer states or are oligomers whose formation is induced by SDS treatment, which has been observed for other proteins [29–30].

To characterize these oligomer states of native protein, analytical size exclusion chromatography data were collected (Figure 2B). As previously reported, *Vs* NnlA exhibited two major peaks, a lower molecular weight peak consistent with a dimer and a second peak consistent with a large oligomer [21]. By contrast, the chromatograms of the purified *Ps*, *Mr*, *Ms*, and *Pd* NnlA were dominated by a single peak ranging in molecular mass from 35.9 to 49.0 kDa, masses consistent with dimers (Table S2 in Supporting Information File 1). The higher oligomer peak was absent in all of these samples. As observed for *Vs* NnlA, there is no evidence for a significant population of monomer in any of these samples. From these data we conclude that these homologs exist mostly as dimers in solution.

**Figure 3:**
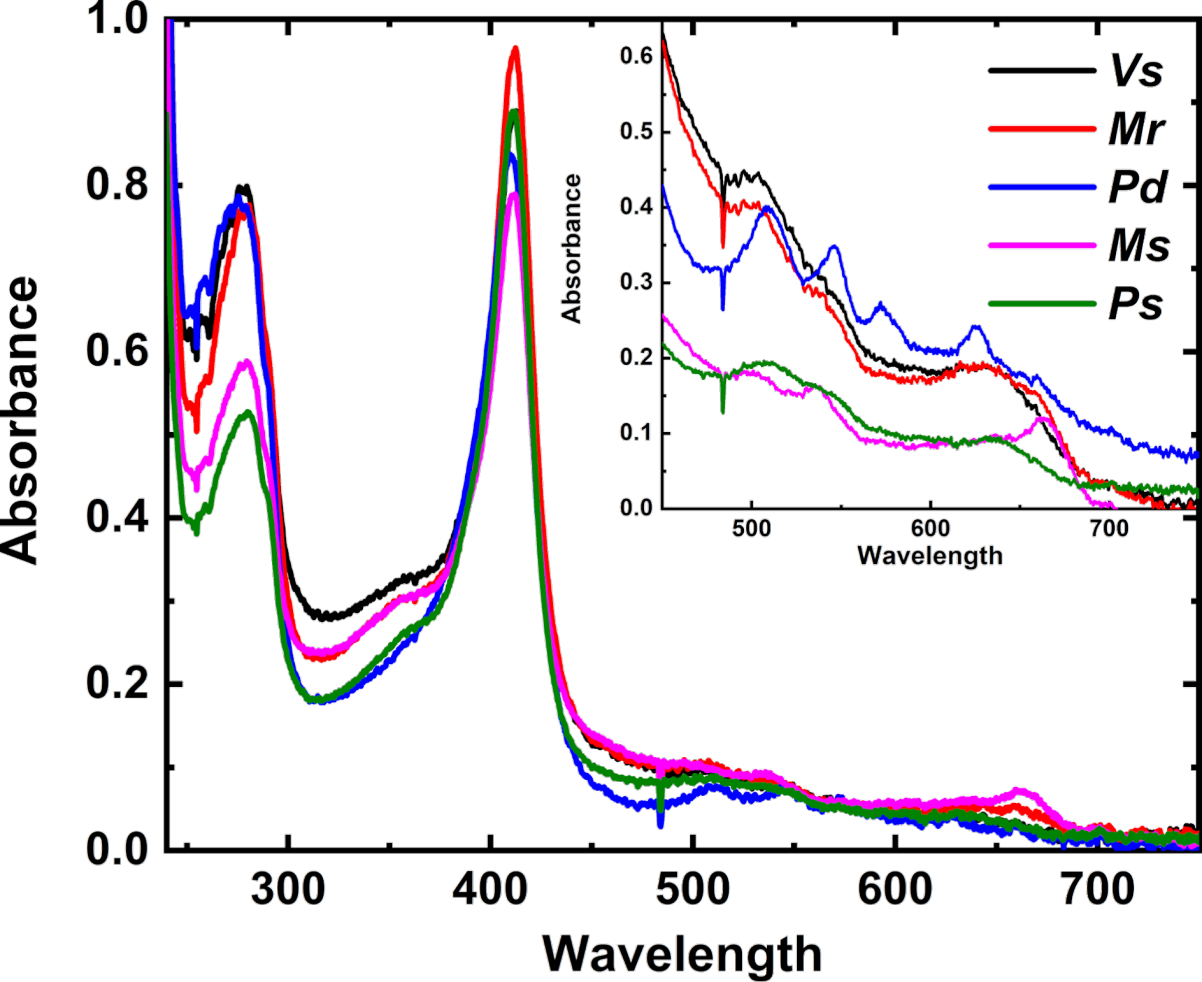
UV–vis absorption spectra of purified NnlA homologs. All spectra were measured in 100 mM tricine, 100 mM NaCl buffer at pH 7.5. NnlA homolog concentrations were *Vs* (10 μM), *Mr* (19 μM), *Pd* (18 μM), *Ms* (13 μM), and *Ps* (4 μM). Inset: Q-band region of the UV–vis spectra for concentrated NnlA homolog samples: *Vs* (90 μM), *Mr* (170 μM), *Pd* (160 μM), *Ms* (38 μM), and *Ps* (28 μM).

Next, the heme incorporation of the isolated homologs was measured. UV–vis absorption spectra showed that each NnlA homolog exhibited characteristic Soret absorption features consistent with heme binding to the protein (Figure 3). In addition, the A_412 nm_/A_280 nm_ ratio for each homolog was greater than 1.0, consistent with high occupancy of heme incorporation in the proteins. Iron analyses of each of the homologs were consistent with this conclusion; the heme iron concentrations per protein were consistent with stoichiometric or nearly stoichiometric heme occupancy (Table 1).

**Table 1:** Iron analyses of purified NnlA homologs.

Sample	[Fe] (μM)	[NnlA] (μM)	[Fe] / [NnlA]

*Vs*	260 ± 30	300 ± 60	0.87 ± 0.21^a^
*Ms*	22.7 ± 1.8	16.7 ± 0.5	1.36 ± 0.08
*Mr*	141.8 ± 12.6	168.8 ± 12.1	0.84 ± 0.10
*Pd*	71.9 ± 12.1	158.9 ± 6.1	0.45 ± 0.08
*Ps*	67.4 ± 11.2	70.7 ± 0.6	0.95 ± 0.16

^a^Ref. [21]

### All homologs degrade NNG to glyoxylate, NH_4_^+^, and NO_2_^−^

**Figure 4:**
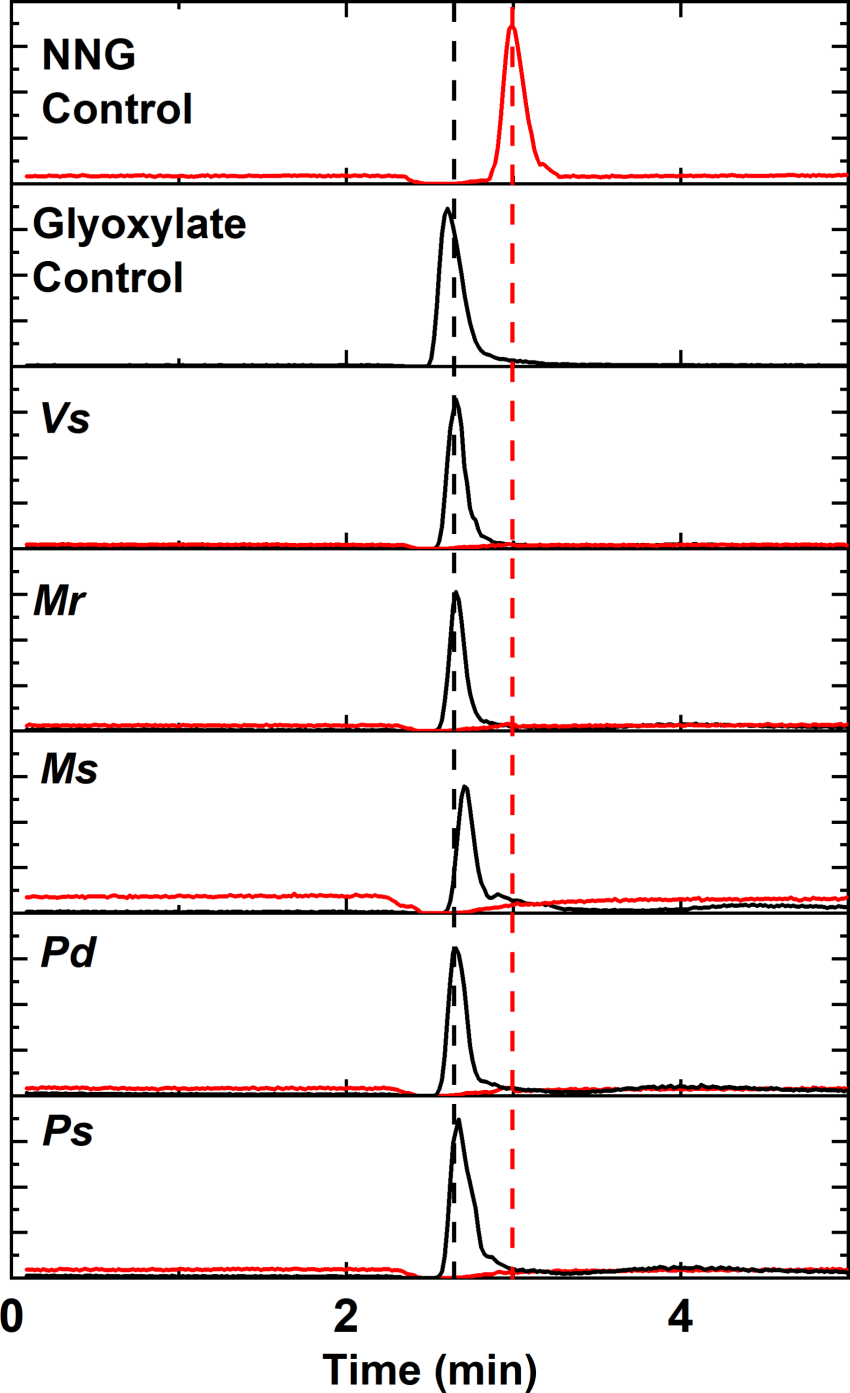
Representative LC–MS EICs monitoring molecular anions of NNG (*m*/*z* 119.01 ± 100 ppm) and glyoxylate (*m*/*z* 72.99 ± 100 ppm) in samples containing 350 μM NNG, 10 μM dithionite, and 5 μM of indicated NnlA homolog. Samples were incubated for approximately one hour at 21 °C in deoxygenated 30 mM tricine buffer at pH 7.5.

Previous work showed that reduction of the *Vs* NnlA heme was required to activate NNG degradation. To test this requirement for the homologs, reduced samples of 5 μM of each NnlA homolog containing 350 μM NNG in deoxygenated 30 mM tricine buffer at pH 7.5 were incubated for one hour at 21 °C in an anaerobic glove box. The samples were analyzed by LC–MS to measure final glyoxylate and NNG concentrations. The extracted ion chromatograms (EICs) showed that NNG was completely consumed and glyoxylate accumulated within the incubation time (Figure 4).

The nitrogenous products in these samples were quantified by enzymatic and colorimetric assays to verify the nitrogen mass balance (Table 2). The data show that nearly stoichiometric concentrations of NH_4_^+^ and NO_2_^−^ are produced per mole of NNG as previously reported for *Vs* NnlA. Samples containing the as-isolated NnlA homologs without any reductant produced negligible concentrations of NH_4_^+^ and NO_2_^−^ (Table S3 in Supporting Information File 1). The combined results indicate each of the four purified NnlA homologs require reduction of the heme cofactor to initiate degradation of NNG to glyoxylate, NH_4_^+^, and NO_2_^−^.

**Table 2:** Nitrogen mass balance resulting from NNG degradation by NnlA.

NnlA^a^	[NNG]_final_ (µM)	[NH_4_^+^]_final_ (µM)	[NO_2_^−^]_final_ (µM)

*Vs*	ND	270 ± 30	240 ± 10
*Mr*	ND	300 ± 30	250 ± 10
*Pd*	ND	290 ± 30	260 ± 10
*Ps*	ND	320 ± 10	250 ± 10
*Ms*	ND	260 ± 10	250 ± 20

^a^Reaction conditions: 5 μM NnlA, 10 µM sodium dithionite, 350 μM NNG in 30 mM tricine buffer at pH 7.5 and room temperature in anaerobic glovebox. Mean values with standard deviations from triplicate reactions are shown.

### NnlA homologs do not degrade 2-NAE

Given the similarities in structure between NNG and 2-NAE, we sought to test if NnlA could also degrade 2-NAE. To test if NnlA could degrade 2-NAE, *E. coli* transformed with *Vs, Mr, Pd, Pj, Ps,* or *Ms* NnlA were incubated overnight at 37 °C in diluted LB containing 300 µM 2-NAE. Post-incubation treatment of the samples with Griess assay revealed that all the cultures lacked NO_2_^−^ (Figure S3, Supporting Information File 1). Combined, these results show that none of the NnlA homologs can degrade 2-NAE.

To ensure the lack of 2-NAE degradation was unrelated to *E. coli* being unable to uptake 2-NAE, in vitro experiments with purified, reduced *Vs* NnlA incubated with 2 mM 2-NAE at pH 7.5 were performed. The LC–MS EICs monitoring 2-NAE (*m*/*z* 105.03 ± 100 ppm) show the prominent peak characteristic for 2-NAE. The intensity of this peak does not change in samples containing reduced *Vs* NnlA compared with samples without *Vs* NnlA. Additionally, NO_2_^−^ was not formed in these samples (Table S4, Supporting Information File 1).

### Alpha-fold model of NnlA

**Figure 5:**
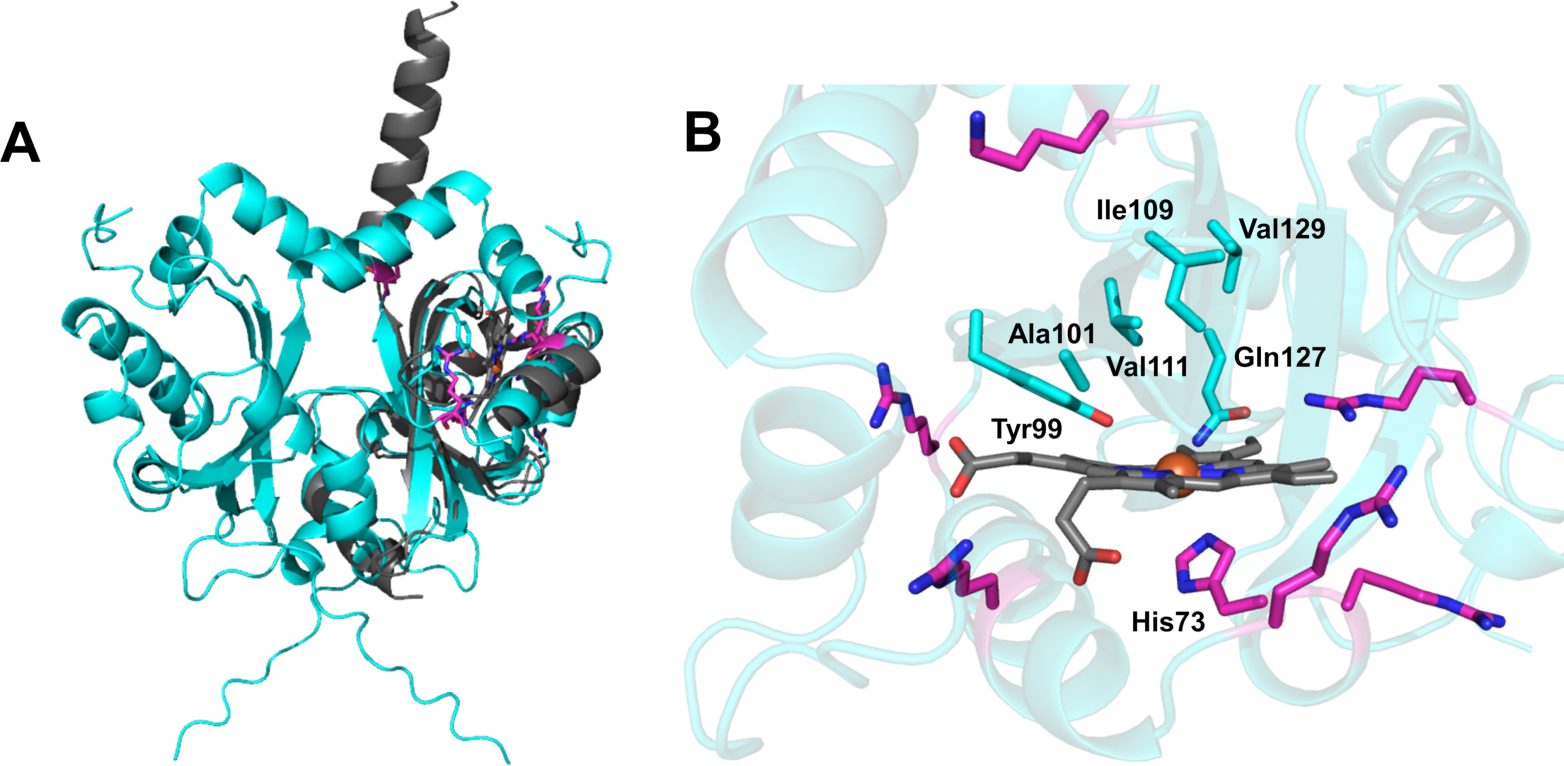
Alpha-fold model of *Vs* NnlA dimer (cyan) overlayed with *Pseudomonas aeruginosa* (grey; PDB: 3VOL [35–36]) to estimate the position and orientation of the heme cofactor. Conserved residues in the distal heme pocket are labeled. Conserved basic residues outside of the heme pocket are colored in magenta. Nitrogen, oxygen, and iron atoms are colored blue, red, and orange, respectively. Figure generated using PyMOL.

A model of the *Vs* NnlA dimer was produced using AlphaFold2, which allows for predicting the structure of oligomeric proteins [31]. The highest ranked model is shown in Figure 5. AlphaFold predicted a canonical α/β fold with high confidence, between residues Arg16 and Gly146 (Figure 5A). Structural clustering using Foldseek Cluster identified substantial similarities to other PAS domain proteins [32].

We sought to identify the heme binding site, but AlphaFold does not model this. However, this AlphaFold model was predicted to bind a heme cofactor by the consensus modeling tool COACH [33]. This protein–ligand model exhibited steric clashes with the heme and protein side chains (data not shown), limiting the use of this model to predict the heme environment. Nevertheless, this protein–ligand model heme binding between the β-sheet and an α-helix based on similarity to the oxygen-sensing dimeric DosH protein [34]. DosH is also a heme-binding PAS-domain containing protein, further validating the assignment of NnlA as a heme-binding PAS domain protein.

As previously reported for a structural homology model of *Vs* NnlA, the heme position was estimated by overlaying the AlphaFold model with the structure of *Pseudomonas aeruginosa* Aer2 (Figure 5B). By this method, the His73 is located near the heme, likely acting as its proximal ligand. As described above, the H73A *Vs* NnlA variant lacked NNG degradation activity and had reduced iron content [21]. The AlphaFold model also predicts a distal pocket composed of Tyr99, Ala101, Ile109, Val111, and Gln127. These amino acids are conserved in an alignment of orthologous protein sequences and could facilitate NNG hydrolysis in the active site (Figure S4, Supporting Information File 1). The function of these residues are being investigated. There are also several nearby conserved basic residues. The significance of these residues will be further discussed below. Other conserved positions could be required for subunit association in the active homodimer.

## Discussion

The combined activity and characterization data indicate that each of the five homologs were similar to *Vs* NnlA in terms of oligomerization (Figure 2B), heme occupancy (Table 1 and Figure 3), NNG degradation activity (Figure 1, Figure 4 and Table 2) and the requirement for heme reduction to initiate NnlA activity (Table S3 in Supporting Information File 1). These similar protein characteristics persist despite the wide range of amino acid sequence identity between the tested homologs (46 to 76%). Therefore, NnlA is unlikely to have recently evolved to exploit anthropogenic nitramine contaminants in a similar fashion as RDX [16,28].

**Figure 6:**
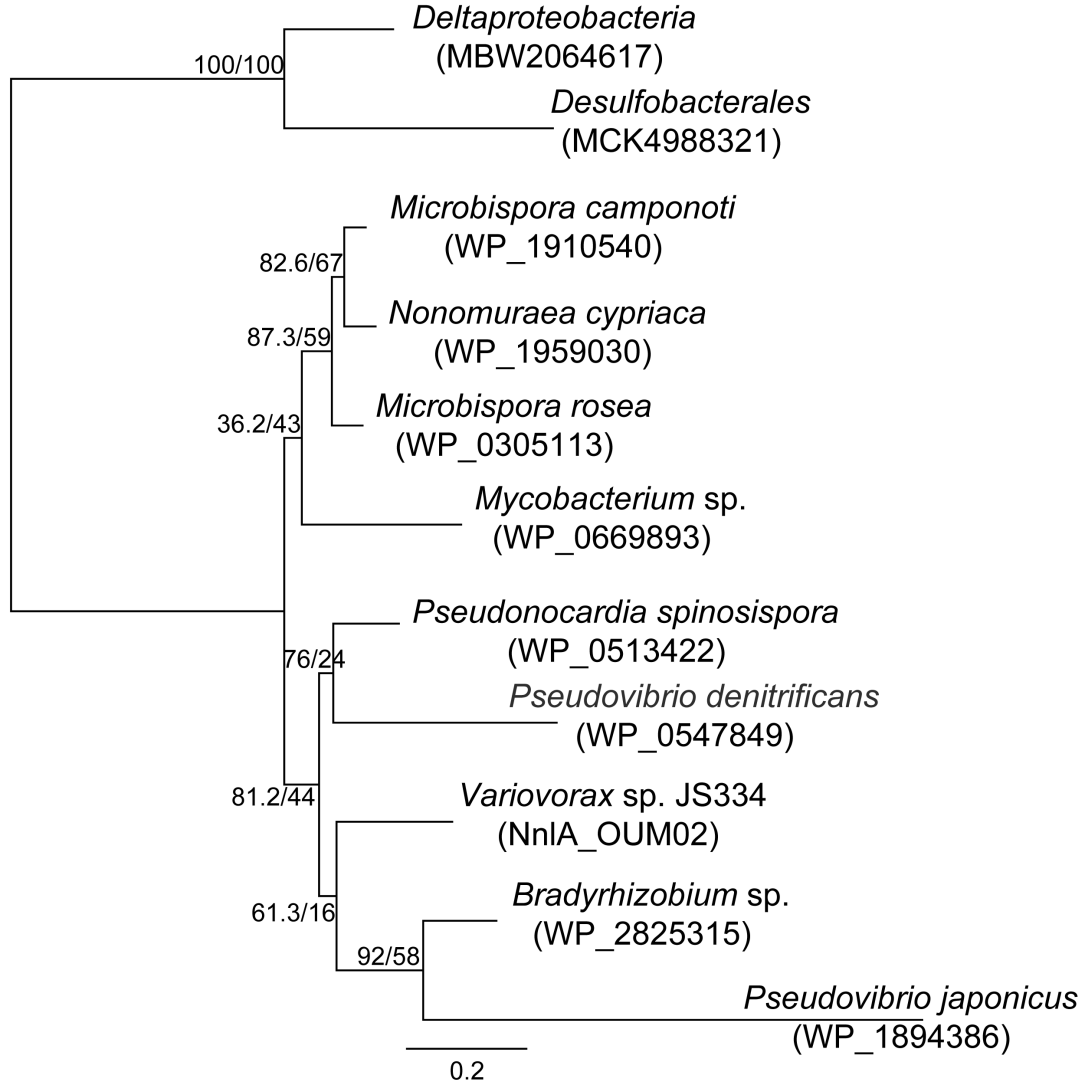
Phylogenetic tree of NnlA homologs with accession numbers. Branch lengths correspond to amino acid substitutions per position. Numbers at nodes indicate Shimodaira–Hasegawa-like approximate likelihood ratio test (SH-aLRT) support (%) and ultrafast bootstrap support (%).

With evidence supporting that all of the homologs were able to degrade NNG, homologs of the *Variovorax nnlA* gene, including those described here, were identified by sequence similarity searches and used to infer phylogenetic relationships. An alignment of 11 amino acid sequences with 28 to 87% identity was prepared resulting in a maximum likelihood tree (Figure 6) [37]. This tree identifies clusters of sequences within taxonomic lineages, suggesting that the gene has been laterally transferred several times, within and among the Alphaproteobacteria, Betaproteobacteria, Deltaproteobacteria, and Actinomycetes lineages. The combined data suggest that NNG degradation activity is found in diverse bacteria. Additionally, these bacteria were isolated from geographically distinct locations (Table S5 in Supporting Information File 1). These results strongly suggest that NnlA, and therefore NNG degradation activity is widespread amongst bacteria and in the environment.

There is no apparent conservation in the gene neighborhoods surrounding the NnlA homologs (Figure S5, Supporting Information File 1). Notably, the YjgF protein previously proposed to aid in deamination of imines – a proposed direct product of NNG degradation by NnlA – is absent [20]. This observation suggests this protein is not needed to aid in glyoxylate formation. Therefore, imine hydrolysis to glyoxylate may occur non-enzymatically or is catalyzed within the NnlA active site. However, an imine product has not yet been observed and further investigations of the NNG degradation mechanism are needed.

Given the potential widespread presence of NnlA, it is possible that NnlA could mediate previously reported nitramine biodegradations. However, prior work showed that NnlA was incapable of degrading nitroguanidine, cyclic nitramines (RDX, HMX), and the linear nitramines, NDAB, and *N*-nitroethylenediamine. As discussed above, several linear nitramines with hydroxy groups were shown to be biodegraded, including the carbon capture byproduct 2-NAE [19]. However, our results indicate that 2-NAE was not degraded either in our cell assays or overnight in the presence of isolated reduced NnlA (see Supporting Information File 1, Figure S3 and Table S4). Therefore, NnlA appears to be specific for NNG degradation.

It is somewhat surprising that NnlA cannot degrade 2-NAE given its structural similarities with NNG. The structural difference between the two molecules is the replacement of the α-carboxylate of NNG with a hydroxy group in 2-NAE. While a decrease in substrate affinity might be expected, there was no activity even in the presence of millimolar concentrations of 2-NAE (Table S4, Supporting Information File 1). This observation suggests that binding in the substrate pocket is dependent on an electrostatic interaction with the NNG α-carboxylate, most likely from a lysine, arginine, histidine side chain or the N-terminus. An alignment of orthologous protein sequences reveals several conserved basic residues (Figure S4, Supporting Information File 1). The only conserved histidine residue is H73, which has been assigned as a heme ligand. Conserved arginine and lysine residues are colored magenta in the *Vs* NnlA model in Figure 5A. Several of these residues are nearby the predicted heme binding site, which may suggest their importance in NNG binding near the heme. However, many of these residues are either far from the heme or would not orient NNG towards the distal pocket of the heme. Future mutagenesis and kinetic experiments or crystallization of the active homodimer will be required to resolve the catalytic mechanism.

**Scheme 2:**
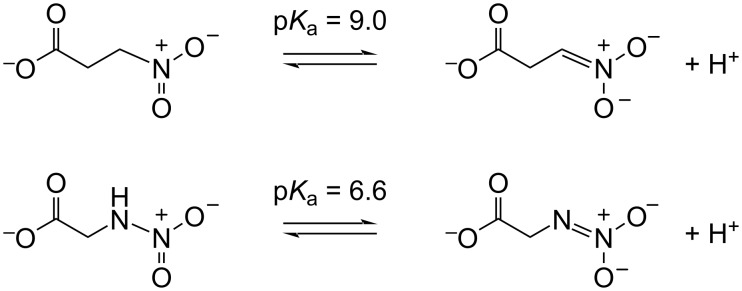
Acid–base equilibrium of 3-nitropropionate (3-NP) vs *N*-nitroglycine (NNG).

If NnlA is specific for NNG as suggested by these results, it is worth speculating about potential functions of NNG and other nitramine natural products. Bacterial natural products often exhibit antibiotic activity and it has been shown that NNG exhibits antibiotic activity towards Gram-negative bacteria (0.18 to 25 μg/mL) [24]. Moreover, the nitramine functional group has potential to serve as a potent warhead in an antibiotic. For example, a cytochrome P450 homolog, XplA, reductively decomposes the nitramine functionality of RDX to form ^•^NO_2_ [38], a toxic reactive nitrogen species. Additionally, NNG is a structural analog of another natural product 3-nitropropionate (3NP) found in plants and fungi [39]. This highly toxic compound inhibited succinate dehydrogenase and other metabolic enzymes. In addition, it has been shown to irreversibly inhibit isocitrate lyase 1 (ICL1) from *Mycobacterium tuberculosis* [40], and key metabolic protein for these pathogens [41]. Isocitrate lyases convert isocitrate to glyoxylate and succinate. Deprotonation of 3NP (p*K*_a_ = 9.0) results in the formation of propionate 3-nitronate (P3N) as a conjugate base (Scheme 2) [39]. It is P3N that directly reacts with a cysteine in the ICL1 active site, forming a thiohydroxamate adduct that inhibits ICL1 turnover [40]. Additionally, the nitronate form of nitro acids has been proposed to behave as a transition analog of carboxylate groups, resulting in nitro compounds also acting as tight-binding reversible inhibitors [42]. Deprotonation of NNG also results in formation of the corresponding nitronate, albeit with a p*K*_a_ of 6.6 [24], far lower than that for 3-NP (Scheme 2). Therefore, a much larger portion of NNG would be expected to exist as the inhibitory nitronate form at physiological pH, suggesting another potential role for nitramine groups as potent warheads in antibiotics.

This antibiotic activity may also require further modification of NNG or its incorporation into a larger natural product. NNG is a non-proteinogenic amino acid, similar to other such N–N containing compounds such as piperazic acid and hydrazinoacetic acid [43]. These precursors are incorporated into larger NPs by non-ribosomal peptide synthases or polyketide synthases, and NNG may have a similar fate [44–45].

Another possibility is that NNG has several physiological functions and fates. For example, a natural product nitronate intermediate was recently shown to have two fates within *Streptomyces achromogenes var. streptozoticus* NRRL 3125 [46]. This nitronate intermediate was shown to be *O*-methylated to form *O*-methylnitronate, and subsequently incorporated into enteromycin. Alternatively, the intermediate could be denitrified by a nitronate monooxygenase (NMO) to produce NO_2_^−^. NnlA could replace NMO as the denitrifying enzyme in NNG producing bacteria, however, a BLAST search of NnlA in the genome of *Streptomyces noursei*, an NNG-producing bacterium, did not reveal any NnlA homologs. Interestingly, four NMOs are annotated in the *S. noursei* genome. These enzymes could protect *S. noursei* from NNG toxicity during its biosynthesis. Meanwhile, we posit that NnlA protects non-NNG producing bacteria from exposure. In vivo experiments comparing the toxicity of NNG towards wild-type cells expressing NnlA and NnlA knockout mutant strains would test this hypothesis.

## Conclusion

In this study the NNG degradation activity of five *Vs* NnlA homologs was screened in *E. coli* transformants, providing evidence that all five degrade NNG. Of these, four were fully isolated and characterized. Each isolated homolog exhibited similar oligomerization and heme occupancy as *Vs* NnlA. In addition, we confirmed by in vitro assays that initiation of NNG degradation activity by the NnlA requires reduction of the heme, verifying the necessity of the heme for NnlA activity. The nitrogen mass balance was consistent with NNG degradation to NO_2_^−^ and NH_4_^+^ as shown for *Vs* NnlA. It was also shown that NnlA cannot degrade the hydroxylated linear nitramine, 2-NAE. The combined data indicate that NnlA homologs specific for NNG degradation activity are found in diverse bacteria and environments. These results suggest the natural product NNG may also be found in diverse environments. The reactivity of the nitramine functionality begs for further studies confirming the natural abundance and physiological functions of NNG and other nitramine natural products.

## Experimental

### General reagents and protocols

Isopropyl β-ᴅ-1-thiogalactopyranoside (IPTG) and 5-aminolevulinic acid (5-ALA) were purchased from Gold Biotechnology. NNG was purchased from AAblocks. 2-NAE was purchased from Toronto Research Chemicals. General buffers and media components were purchased from Fisher Scientific or VWR. Stock dithionite concentrations were determined by UV–vis absorbance at 318 nm (ε_318_ = 8000 M^−1^cm^−1^). Water used for all solutions was of 18.2 MΩ·cm resistivity from a Barnstead Nanopure (Thermo Fisher Scientific). Solvents for LC–MS experiments were of at least HPLC grade and contained 0.1% vol/vol formic acid.

### Protein expression and purification

The vectors to express *Vs* NnlA and H73A *Vs* NnlA were previously reported [21]. *Pd*, *Mr*, *Ms*, *Ps*, and *Pj nnla* genes were synthesized as *E. coli* codon-optimized constructs and cloned into the NdeI and XhoI restriction sites of pET-28a(+)-TEV by GenScript.

For protein expression, plasmids were electroporated into *E. coli* BL21(DE3) cells and protein was expressed and purified by immobilized metal affinity chromatography (IMAC) as previously described for *Vs* NnlA [21]. The only modification was that plasmids using pET-28a(+)-TEV required 50 µg/mL kanamycin instead of 100 µg/mL ampicillin. IMAC purified and concentrated protein were exchanged into 100 mM tricine, 100 mM NaCl buffer at pH 7.5 and stored at −60 °C.

### Protein characterization

Total iron concentrations in protein samples were quantified using an iron assay that allows for release and subsequent detection of heme-ligated iron [47]. Protein concentration was determined using bicinchoninic acid protein quantification assay (Pierce). The oligomeric state was determined by processing the protein through Superdex 200 Increase 10/300 GL analytical size exclusion column with 100 mM tricine with 100 mM NaCl at pH 7.5 as the mobile phase. Protein size exclusion chromatography standards (BioRad) were used to determine molecular masses.

### Nitramine degradation assays

LC–MS analysis was performed using an Agilent 1260 LC stack equipped with a Zorbax RX-C18 column (5 μm, 4.6 × 150 mm) and connected to an Agilent 6230 TOF mass spectrometer with electrospray ionization (ESI). Analyses used an isocratic mixture containing 65% water, 25% acetonitrile, and 10% isopropanol at a flow rate of 0.5 mL/min. The mass spectrometer was run in the negative ion mode with a probe voltage of 4,500 V and a fragmentation voltage of 175 V. To monitor NNG, 2-NAE, and glyoxylate, extracted ion chromatograms were obtained at *m*/*z* 119.0, 105.0, and 73.0, respectively.

Ammonium concentrations were determined using a glutamate dehydrogenase assay (Sigma-Aldrich) kit using the manufacturer’s instructions. Nitrite concentrations were determined by reacting 25 μL aliquots of reaction sample with 25 μL of deoxygenated Griess reagent R1 (1% sulfanilamide in 5% H_3_PO_4_) followed by addition of 25 μL of deoxygenated Griess reagent R2 (0.1% naphthylethylenediamine dihydrochloride in water). The absorbance was read at 548 nm using an Infinite M200 Plate Reader (Tecan). Nitrite concentrations were determined by comparison of A_548 nm_ to a nitrite standard curve.

### Screening of *E. coli* transformants for NNG or 2-NAE degradation activity

Transformation of NnlA homologs were obtained as described above. The cells were then plated on LB agar plates containing ampicillin (100 µg/mL) or kanamycin (50 µg/mL) as appropriate and incubated overnight at 37 °C. Three colonies from each plate were picked with a toothpick and then resuspended in 0.2 mL of sterile water. Thirty microliter aliquots of suspended cells were used to inoculate 100 µL of selective growth media (1/5 LB media, 20 µM isopropyl β-ᴅ-thiogalactopyranoside (IPTG), 300 µM 2-NAE or 3 mM NNG, and antibiotic) in a 96-well plate. After overnight incubation at 37 °C, the cells were pelleted by centrifugation and the nitrite quantified by Griess assay as described above.

### Preparation of NNG and 2-NAE degradation samples

Triplicate samples containing 2 mM 2-NAE or 350 µM NNG, 40 µM titanium citrate with or without 20 µM *Vs* NnlA in deoxygenated 23 mM tricine at pH 7.5 were incubated overnight at 21 °C.

### Phylogenetic tree

Homologs of the *Variovorax nnlA* gene, including those described here, were identified by sequence similarity searches and their predicted amino acid sequences were used to infer phylogenetic relationships. An alignment of 11 amino acid sequences with 28 to 87% identity was prepared using MUSCLE software (ver. 5.1) [48] and trimmed to 148 positions in conserved blocks using Gblocks (ver. 0.91b) [49]. A maximum likelihood tree was inferred using IQ-TREE (ver. 2.2.2.6) with the LG+G4 substitution model [37].

## Supporting Information

File 1 Additional Figures and Tables.

## Data Availability

The data that supports the findings of this study is available from the corresponding author upon reasonable request.

## References

[1] Crocker F H, Indest K J, Fredrickson H L (2006). Appl Microbiol Biotechnol.

[2] Pichtel J (2012). Appl Environ Soil Sci.

[3] Abadin H, Ingerman L, Smith C (2012). Toxicological profile for RDX.

[4] Bachmann W E, Sheehan J C (1949). J Am Chem Soc.

[5] Lapointe M-C, Martel R, Diaz E (2017). J Environ Qual.

[6] D’Amico L, Blessinger T, Subramaniam R (2018). Toxicological Review of Hexahydro-1,3,5-trinitro-1,3,5-triazine (RDX).

[7] Maleh H K, Carvalho-Knighton K M, Martin D F (2009). Fla Sci.

[8] Stone W J, Paletta T L, Heiman E M, Bruce J I, Knepshield J H (1969). Arch Intern Med.

[9] Balakrishnan V K, Halasz A, Hawari J (2003). Environ Sci Technol.

[10] Chatterjee S, Deb U, Datta S, Walther C, Gupta D K (2017). Chemosphere.

[11] Fournier D, Halasz A, Thiboutot S, Ampleman G, Manno D, Hawari J (2004). Environ Sci Technol.

[12] Sabir D K, Grosjean N, Rylott E L, Bruce N C (2017). FEMS Microbiol Lett.

[13] Låg M, Lindeman B, Instanes C (2011). Health effects of amines and derivatives associated with CO2 capture.

[14] Yu K, Mitch W A, Dai N (2017). Environ Sci Technol.

[15] Paquet L, Monteil-Rivera F, Hatzinger P B, Fuller M E, Hawari J (2011). J Environ Monit.

[16] Rylott E L, Jackson R G, Sabbadin F, Seth-Smith H M B, Edwards J, Chong C S, Strand S E, Grogan G, Bruce N C (2011). Biochim Biophys Acta, Proteins Proteomics.

[17] Fournier D, Halasz A, Spain J, Spanggord R J, Bottaro J C, Hawari J (2004). Appl Environ Microbiol.

[18] Fournier D, Trott S, Hawari J, Spain J (2005). Appl Environ Microbiol.

[19] Brakstad O G, Sørensen L, Zahlsen K, Bonaunet K, Hyldbakk A, Booth A M (2018). Int J Greenhouse Gas Control.

[20] Mahan K M, Zheng H, Fida T T, Parry R J, Graham D E, Spain J C (2017). Appl Environ Microbiol.

[21] Strickland K A, Holland A A, Trudeau A, Szlamkowicz I, Beazley M J, Anagnostopoulos V A, Graham D E, Caranto J D (2022). Appl Environ Microbiol.

[22] Parry R, Nishino S, Spain J (2011). Nat Prod Rep.

[23] Graham D E, Spain J C, Parry R J (2018). Nitration Enzyme Toolkit for the Biosynthesis of Energetic Materials.

[24] Miyazaki Y, Kono Y, Shimazu A, Takeuchi S, Yonehara H (1968). J Antibiot.

[25] Alston T A, Seitz S P, Porter D J T, Bright H J (1980). Biochem Biophys Res Commun.

[26] Borer K, Hardy R, Lindsay W, Spratt D, Mees G (1966). J Exp Bot.

[27] Andeer P F, Stahl D A, Bruce N C, Strand S E (2009). Appl Environ Microbiol.

[28] Chong C S, Sabir D K, Lorenz A, Bontemps C, Andeer P, Stahl D A, Strand S E, Rylott E L, Bruce N C (2014). Appl Environ Microbiol.

[29] Matsui T, Kamata S, Ishii K, Maruno T, Ghanem N, Uchiyama S, Kato K, Suzuki A, Oda-Ueda N, Ogawa T (2019). Sci Rep.

[30] Verhagen M F J M, Voorhorst W G B, Kolkman J A, Wolbert R B G, Hagen W R (1993). FEBS Lett.

[31] Mirdita M, Schütze K, Moriwaki Y, Heo L, Ovchinnikov S, Steinegger M (2022). Nat Methods.

[32] Barrio-Hernandez I, Yeo J, Jänes J, Mirdita M, Gilchrist C L M, Wein T, Varadi M, Velankar S, Beltrao P, Steinegger M (2023). Nature.

[33] Yang J, Roy A, Zhang Y (2013). Bioinformatics.

[34] Park, Suquet C, Satterlee J D, Kang C (2004). Biochemistry.

[35] Sawai H, Sugimoto H, Shiro Y, Aono S X-ray Crystal Structure of PAS-HAMP Aer2 in the CN-bound Form.

[36] Sawai H, Sugimoto H, Shiro Y, Ishikawa H, Mizutani Y, Aono S (2012). Chem Commun.

[37] Minh B Q, Schmidt H A, Chernomor O, Schrempf D, Woodhams M D, von Haeseler A, Lanfear R (2020). Mol Biol Evol.

[38] Halasz A, Manno D, Perreault N N, Sabbadin F, Bruce N C, Hawari J (2012). Environ Sci Technol.

[39] Francis K, Smitherman C, Nishino S F, Spain J C, Gadda G (2013). IUBMB Life.

[40] Ray S, Kreitler D F, Gulick A M, Murkin A S (2018). ACS Chem Biol.

[41] McKinney J D, zu Bentrup K H, Muñoz-Elías E J, Miczak A, Chen B, Chan W-T, Swenson D, Sacchettini J C, Jacobs W R, Russell D G (2000). Nature.

[42] Alston T A, Porter D J T, Bright H J (1983). Acc Chem Res.

[43] Hedges J B, Ryan K S (2020). Chem Rev.

[44] Wei Z-W, Niikura H, Morgan K D, Vacariu C M, Andersen R J, Ryan K S (2022). J Am Chem Soc.

[45] Morgan K D, Andersen R J, Ryan K S (2019). Nat Prod Rep.

[46] He H-Y, Ryan K S (2021). Nat Chem.

[47] Fish W W (1988). Methods Enzymol.

[48] Edgar R C (2004). BMC Bioinf.

[49] Castresana J (2000). Mol Biol Evol.

