# Supplementary material for: Activity assays of NnlA homologs suggest the natural product N-nitroglycine is degraded by diverse bacteria
Source: Beilstein J Org Chem. 2024 Apr 17;20:830–40. doi: 10.3762/bjoc.20.75 (PMC11035981; doi:10.3762/bjoc.20.75)
Supplement: File 1 — Additional Figures and Tables. [file Beilstein_J_Org_Chem-20-830-s001.pdf]

BEILSTEIN JOURNAL OF ORGANIC CHEMISTRY

# Supporting Information

for

# Activity assays of NnlA homologs suggest the natural product *N*-nitroglycine is degraded by diverse bacteria

Kara A. Strickland, Brenda Martinez Rodriguez, Ashley A. Holland, Shelby Wagner, Michelle Luna-Alva, David E. Graham and Jonathan D. Caranto

*Beilstein J. Org. Chem.* **2024,** *20,* 830–840. doi:10.3762/bjoc.20.75

# Additional Figures and Tables

License and Terms: This is a supporting information file under the terms of the Creative Commons Attribution License (https://creativecommons.org/licenses/by/4.0). Please note that the reuse, redistribution and reproduction in particular requires that the author(s) and source are credited and that individual graphics may be subject to special legal provisions.
The license is subject to the *Beilstein Journal of Organic Chemistry* terms and conditions: (https://www.beilstein-journals.org/bjoc/terms)

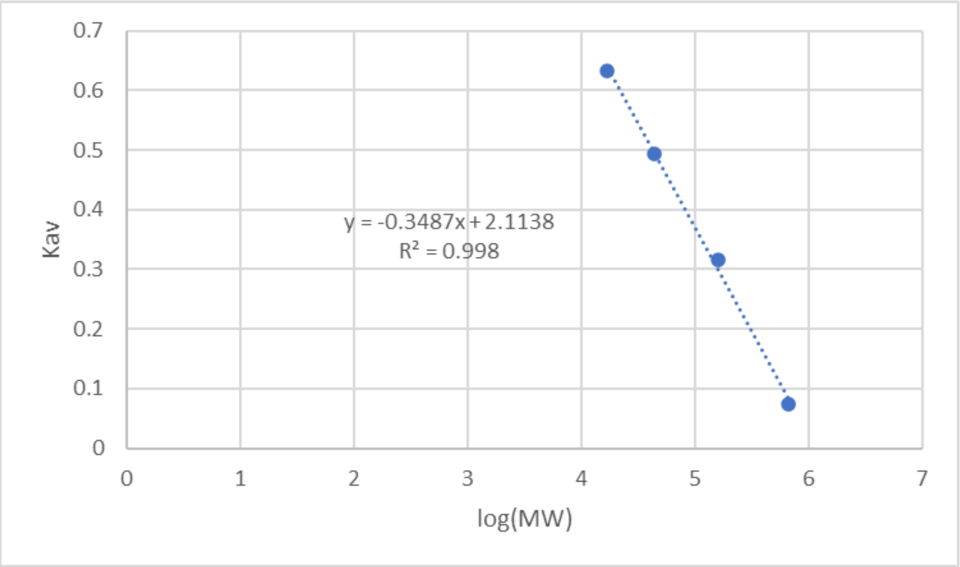


**Figure S1:** Analytical size exclusion chromatography calibration curve of standards. Flow rate 0.75 mL/min, 100 mM tricine and 100 mM NaCl buffer pH 7.5.

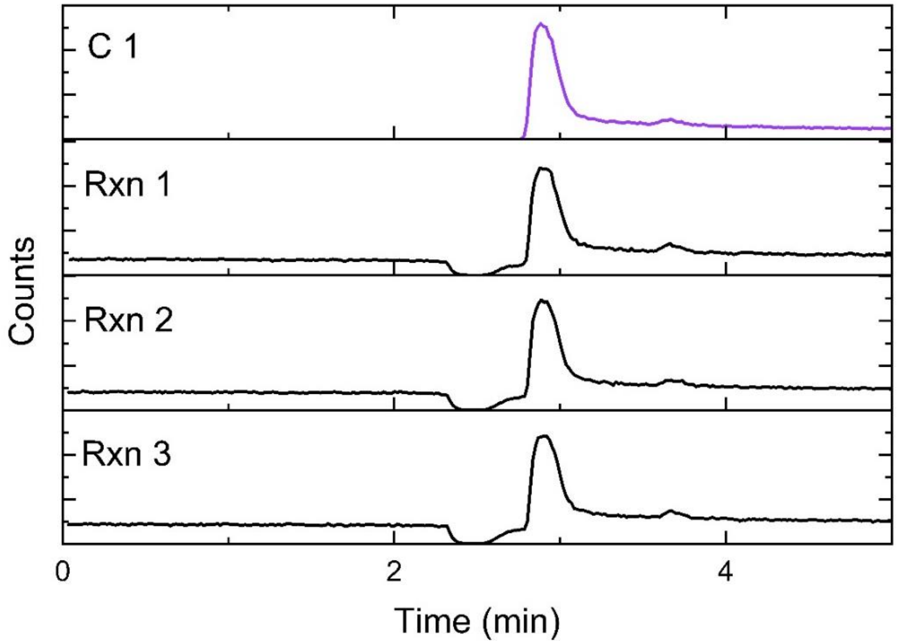


**Figure S2:** Representative LC–MS EICs monitoring molecular anion of 2-NAE (*m/z* 105.03) in samples containing 2 mM 2-NAE, excess titanium citrate, and either no *Vs* NnlA for the control sample or 20 µM reduced *Vs* NnlA ($Fe^{II}$-NnlA) for the reaction samples. Samples were incubated overnight at room temperature in deoxygenated 23.3 mM tricine buffer, pH 7.5.

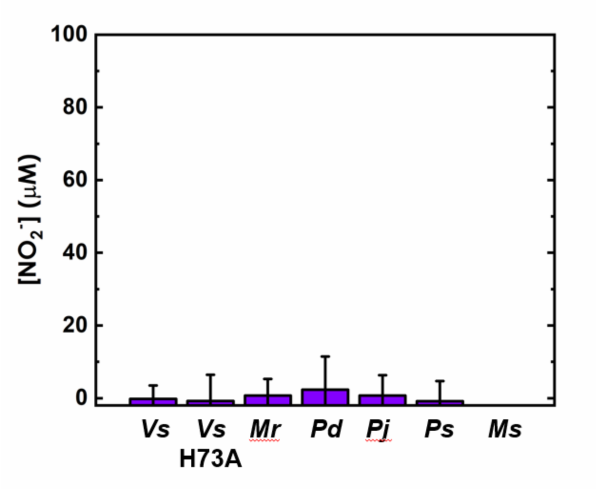


**Figure S3:** Nitrite concentrations observed in overnight cultures of *E. coli* transformed with NnlA homologs or variants grown in the presence of 2-NAE. Cells were incubated overnight in diluted LB containing IPTG and 3 mM NNG and incubated overnight at 37 °C.

```
MBW2064617           --------------MAGNGDKRLTELIRLAMECMGVAVTIIDPQGTLLYYNKQAEKILDR    46
MCK4988321           -------------MNENERKTKLGELVNLAMDCLGVAVTIIDTKGTLLYYNQHSAKILDR    47
WP_189438608         MSQNQHSAFRKQVADRTLDGWELEGCAEWLIDQQGVGVSIIDTEGRLLFYNQWADNKMPR    60
WP_054784913         -MTDNNNELPEVTDQRILEAWKLSGWADRLLEEAGIGVTIIDKDGKLLYYNKWASENLDR    59
WP_282531508         ----MDEKLPEVTQQRVLPGWTVSQWAGGLIEHAGVGVTILDREGRVMFYNQWAANRLDR    56
NnlA_(OUM02170)      MNQVNTEELPEVVDQRILAGWRLSEWADRILEYAGVGVTLVDRLGRCVYYNQWAKDHLDR    60
WP_066989343         --MTTHADLTEVFEHRIVADWALGEWADRLLEQAGLGVTIVDRHGVVMYYNKWAAEHLDR    58
WP_051342206         ---MTQAILPEVTDARILDGWQLSGWADRLLEQAGVGVTIVDRTGRVLYYNKWADEHLDR    57
WP_030511367         --MTSQAEPAEAAESRIATDWGLDEWADRLIDQAGVGVTILNRHGTVMYYNKWASEHLDR    58
WP_191054027         --MSSQVELAEVAESRIATDWGLDQWADRILEQAGFGVTVLDRHGTVMYYNKWASEHLDR    58
WP_195903080         --MSSQVELAEVAESRIATDWGLDQWADRILEQAGVGVTILDRHGTVMYYNKWASEHLDR    58
                                           :       ::  *..*::::  *  ::**: : . : *
```

```
MBW2064617           KPEYIGKDVHSHHKRAASNKKLDMMLEDFQ-KGRTEPFHYQARPYGE-TILVILSPIFED    104
MCK4988321           KPEYIGTDIHSHHKEAAINKKVDLMLKEFE-GGRKDHFHYEAKPYGK-IIFVTLAPIIKN    105
WP_189438608         EPEYLGQKVQEHHRKQITNVRFEAMLDLFRKEGRTEAVKYVAKPYEGLTIIVIVTPIIVE    120
WP_054784913         QPRHIGHNVKENHRRSITNPRFDAMLQLFR-DGRKDPVRYVANPYGTTTILVTVSPIHID    118
WP_282531508         KPEYIGKDVRNHHRRKITNPRFDAMLKLFE-EGRTDPVHYVARPYGKITILVTVSPIKVD    115
NnlA_(OUM02170)      KPGYIGDEIHNRHRRAITNPRFDAMLKLFE-EGRMEPVRYVARPYGKTTILVTVSPIYVE    119
WP_066989343         QPGYLGHSVHERHHRKITNPRFDAMLKLFV-DGRIEPVQYVARPYGKTTILVTVSPIRIG    117
WP_051342206         KPEYIGNDVRDRHRQPITNPRFDAMIALFE-EGRVEPVRYVARPYGKTIILVTVSPIWVD    116
WP_030511367         RPEYIGNDVRKRHRRAVTNPRFDAMLKLFE-DGRVEPVRYVARPYGKTTILVTVSPIRVD    117
WP_191054027         KPEYIGNDVRKRHRRAVTNPRFDAMLRLFE-EGRVEPVRYVARPYGRTTILVTVSPIRVD    117
WP_195903080         MPEYIGNDVRKHHRRAVTNPRFDAMLRLFE-EGRVEPVSYVARPYGKITILVTVSPIRVN    117
                      * ::* .::..*:.   * :.: *:  *   ** : . * *.**    *:* ::**

MBW2064617           AKFVGCVQCVRLKDDTESR--------------------------------------       123
MCK4988321           GEFLGCVQTVRLKNTVSANQ-------------------------------------       125
WP_189438608         GELVAFCQTVLDKDEIQGMCETFDESGNITFQRDILPGSEPG---------------       162
WP_054784913         EELVGFSQFVLLKEEVQELCCLFDQHGRDPFEKDMLPNGPPT---------------       160
WP_282531508         GELVGYSQIVLMKDEIQELFRRFDESGRESFEKDMLPAWPFSGND------------       160
NnlA_(OUM02170)      GELVGYSQIVLLKDEVEALCQRFNASGRESFEREMLPDSTPSNDD------------       164
WP_066989343         GELVGLAQLVLLKDEVQELFSRFDDSGRESFERDMLPDGYPGA--------------       160
WP_051342206         GELVGFSQIVLLKNEVQELCERFDASGRESFEREMLPNGATGY-------LTYKKNT       166
WP_030511367         GELVGFSQIVLLKDEVQELCARFDESGRESFEREMLPNGPPAT--------------       160
WP_191054027         GELVGFSQVVLLKDEIQELCARFDESGRESFEREMLPDTPAVARDPAAGQCSSRRS-       173
WP_195903080         GELVGFSQIVLLKDEVQELFALFDESGRESFEREMLPNGLPTA--------------       160
                      :::.  * *  *:  .
```

**Figure S4:** Amino sequence alignment of NnlA homologs shown in Figure 6 of main text. Conserved basic residues are colored red.

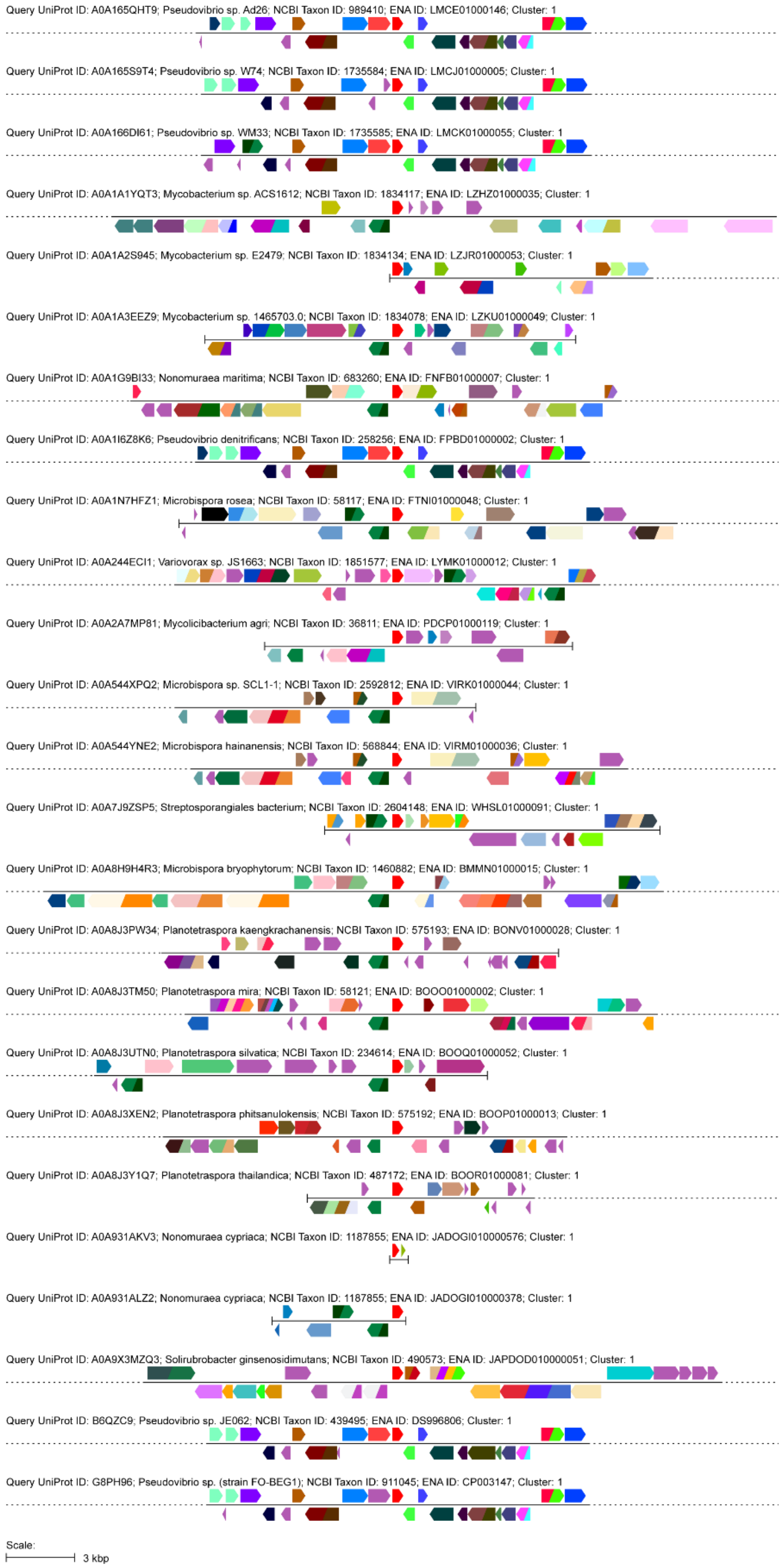


**Figure S5:** Gene neighborhoods of NnlA homologs. NnlA homologs in each neighborhood is color coded red in the middle of the figure.

| **Table S1:** Elution times for standard by size exclusion chromatography.[a] | | | |
|---|---|---|---|
| Standards | MW | Elution volumes | Gel phase distribution coefficient Kav |
| Thyroglobulin | 670000 | 10.19 | 0.073 |
| γ-globulin | 158000 | 13.82 | 0.317 |
| Ovalbumin | 44000 | 16.45 | 0.493 |
| Myoglobin | 17000 | 18.54 | 0.634 |
| [a]Flow rate 0.75 mL/min, 100 mM tricine and 100 mM NaCl buffer pH 7.5. | | | |

| **Table S2:** NnlA homologs analytical size exclusion values.[a] | | | |
|---|---|---|---|
| Protein sample | MW | Elution volume | Gel phase distribution coefficient Kav |
| *Pd* NnlA | 49,000 | 16.23 | 0.478 |
| *Ps* NnlA | 38,200 | 16.79 | 0.516 |
| *Ms* NnlA | 36,400 | 16.90 | 0.523 |
| *Mr* NnlA | 35,900 | 16.93 | 0.526 |
| Oligomer *Vs* NnlA | 397,000 | 11.51 | 0.162 |
| Dimer *Vs* NnlA | 41,400 | 16.61 | 0.504 |
| [a]Flow rate 0.75 mL/min, 100 mM tricine and 100 mM NaCl buffer pH 7.5. | | | |

| **Table S3:** Nitrogen mass balance resulting from NnlA reaction with NNG | | |
|---|---|---|
| **NnlA[a]** | **Reduced NnlA $[NO_2^-]_{final}$ (μM)** | **As Isolated NnlA $[NO_2^-]_{final}$ (μM)** |
| *Mr* | 250 ± 10 | 10.6 ± 1.7 |
| *Pd* | 260 ± 10 | 51.3 ± 5.3 |
| *Ps* | 250 ± 10 | 31.0 ± 6.7 |
| *Ms* | 250 ± 20 | 22.2 ± 8.5 |
| [a]Reaction conditions: 5 μM NnlA, 10 μM sodium dithionite for reduced NnlA and no reducing agent for as isolated NnlA, 350 μM NNG in 30 mM tricine buffer at pH 7.5 and room temperature in anaerobic glovebox. | | |

| **Table S4:** Test of 2-NAE degradation *Vs* NnlA (m/z 105.03).[a] | | |
|---|---|---|
| **Sample** | **$[NO_2^-]_{final}$ (μM)** | **Area of Integration** |
| Control samples | -5.5 ± 1.4 | $2.4 \pm 0.4 \times 10^6$ |
| Reaction samples | 1.8 ± 11.0 | $2.0 \pm 0.5 \times 10^6$ |
| [a]Samples containing 2 mM 2-NAE, excess titanium citrate, and either no *Vs* NnlA for the control samples or 20 μM reduced NnlA ($Fe^{II}$-NnlA) for the reaction samples. Samples were incubated overnight at room temperature in deoxygenated 23.3 mM tricine buffer pH 7.5 | | |

| **Table S5:** Expected NNG degrading bacteria based on this study. | | | |
|---|---|---|---|
| **Species** | **Bacterial class** | **Location isolated** | **Ref.** |
| *Variovorax sp.* Strain *JS 1663* | betaproteobacteria | USA: activated sludge from Ammunition Plant | [1] |
| *Pseudovibrio denitrificans* JCM 12308 | alphaproteobacteria | Taiwan: seawater | [2] |
| *Pseudovibrio japonicus* strain KCTC 12861 | alphaproteobacteria | Japan: seawater | [3] |
| *Pseudonocardia spinosispora* DSM 44797 | actinomycetia | S. Korea: soil | [4] |
| *Mycobacterium sp.* 1465703.0 | actinomycetia | Mozambique: Host cultures | J. Craig Venter Institute Genome Center for Infectious Diseases.Accession: PRJNA305922 |
| *Microbispora rosea subsp. nonnitritogenes* strain NRRL B-2631 | actinomycetia | Unknown: acidic volcanic ash | Ref. [5] |

## References

1. Mahan, K. M.; Zheng, H.; Fida, T. T.; Parry, R. J.; Graham, D. E.; Spain, J. C., A novel, iron-dependent enzyme that catalyzes the initial step in the biodegradation of N-nitroglycine by Variovorax sp. strain JS1663. *Appl. Environ. Microbiol.* **2017,** *83*, e00457-17.
2. Shieh, W. Y., Lin, Y. T., Jean, W. D. Pseudovibrio denitrificans gen. nov., sp. nov., a marine, facultatively anaerobic, fermentative bacterium capable of denitrification. *International journal of systematic and evolutionary microbiology*, **2004**, *54*(6), 2307-2312
3. Hosoya, S., Yokota, A., Pseudovibrio japonicus sp. nov., isolated from coastal seawater in Japan. *International journal of systematic and evolutionary microbiology*. **2007**, 57(9) 1952-1955.
4. Lee, S. D., Kim, E. S., Kang, S. O., Hah, Y. C. Pseudonocardia spinosispora sp. nov., isolated from Korean soil. *International journal of systematic and evolutionary microbiology*, **2002**, *52*(5), 1603-1608.
5. Nonomura, H. Distribution of actinomycetes in the soil. IV. Isolation and taxonomy of the genus Microbispora. *J. Ferment. Technol.* **1960**, *38*, 401-405.
